# Predictors of mortality in COVID-19 patients treated with convalescent plasma therapy

**DOI:** 10.1371/journal.pone.0271036

**Published:** 2022-07-19

**Authors:** Naomi Rahimi-Levene, Jonathan Shapira, Irma Tzur, Eli Shiloah, Victoria Peer, Ella Levin, Marina Izak, Eilat Shinar, Tomer Ziv-Baran, Miriam Weinberger, Oren Zimhony, Jacob Chen, Yasmin Maor

**Affiliations:** 1 Blood Bank, Shamir Medical Center, Zerifin, Israel; 2 Internal Medicine Department H, Shamir Medical Center, Zerifin, Israel; 3 Internal Medicine Department F, Shamir Medical Center, Zerifin, Israel; 4 Internal Medicine Department E, Shamir Medical Center, Zerifin, Israel; 5 National Blood Services, Magen David Adom, Ramat Gan, Israel; 6 Department of Epidemiological Studies, Sackler Faculty of Medicine, Tel Aviv University, Tel Aviv, Israel; 7 Department of Infectious Diseases, Shamir Medical Center, Zerifin, Israel; 8 Sackler Faculty of Medicine, Tel Aviv University, Tel Aviv, Israel; 9 Infectious Diseases Unit, Kaplan Medical Center, Rehovot, Israel; 10 Hospital Management, Meir Medical Center, Kfar Saba, Israel; 11 Trauma and Combat Medicine Branch, Israel Defense Forces, Medical Corps, Tel Hashomer, Ramat Gan, Israel; 12 Department of Infectious Diseases, Wolfson Medical Center, Holon, Israel; Consejo Nacional de Investigaciones Cientificas y Tecnicas, ARGENTINA

## Abstract

Several options to treat hospitalized severe COVID-19 patients have been suggested. The study aimed to describe survival in patients treated with convalescent COVID plasma (CCP) and to identify in-hospital mortality predictors. This prospective cohort study examined data from 112 severe COVID-19 patients hospitalized in the Corona Departments in an acute care hospital who received two units of CCP (at least one of them high-titer). Demographic and medical data was retrieved from the patients’ electronic health records (EHR). Possible predictors for in-hospital mortality were analyzed in a univariate analysis and those found to be clinically significant were further analyzed in a multivariable analysis. Median age was 67 years (IQR 55–74) and 66 (58.9%) of them were males. Of them, 20 (17.9%) died in hospital. On multivariable analysis diabetes mellitus (p = 0.004, OR 91.54), mechanical ventilation (p = 0.001, OR 59.07) and lower albumin levels at treatment (p = 0.027, OR 0.74) were significantly associated with increased in-hospital mortality. In our study, in-hospital mortality in patients receiving CCP is similar to that reported for the general population, however certain variables mentioned above were associated with increased in-hospital mortality. In the literature, these variables were also associated with a worse outcome in patients with COVID-19 who did not receive CCP. As evidence points toward a benefit from CCP treatment in immunocompromised patients, we believe the above risk factors can further define COVID-19 patients at increased risk for mortality, enabling the selection of candidates for early treatment in an outpatient setting if possible.

## Introduction

### Background

Treatment options for severe COVID-19 are being developed and introduced [1]. The use of convalescent plasma is not new, and has been reported in the literature as treatment for a variety of infectious diseases. It was administered to patients with Bolivian hemorrhagic fever [2], Ebola virus [3], CoV-MERS infection [4] and in the treatment of patients with severe acute respiratory syndrome (SARS) [5]. The effect of CCP in COVID-19 patients is complex and is thought to be via anti-SARS-CoV-2 antibodies, particularly neutralizing antibodies. Other factors considered to be beneficial against COVID-19 are non-neutralizing anti-spike antibodies, antithrombin, alpha-1 antitrypsin, ACE-2, albumin and others [6]. Based on published data, CCP in COVID-19 patients appears safe [7]. A multicenter, randomized clinical trial performed in 7 medical centers in Wuhan, China, among patients with severe COVID-19, CCP therapy along with standard treatment did not result in a statistically remarkable improvement [8]. Joyner et al. reported on 5000 hospitalized adults with severe COVID-19 who received CCP safely without excessive mortality [9]. Furthermore, data on 20,000 patients with COVID-19 in USA provided evidence that transfusion of convalescent plasma is safe and earlier administration could reduce mortality [10]. Recent data suggests that this treatment can benefit immunocompromised patients [11–13].

With the knowledge that CCP collected from recovered COVID-19 patients contains antibodies against SARS-CoV-2, we administered CCP to patients with severe disease as part of a national study. The goal of this study was to identify predictors of in-hospital mortality in severe COVID-19 patients who received CCP.

## Methods

### Study design

A prospective cohort study of all patients with severe COVID-19 pneumonia who were hospitalized in the Shamir Medical Center and received CCP.

The Shamir Medical Center is an 891 bed acute care university hospital in central Israel, which treats an urban and rural multiethnic population of above 1.5 million people.

All-cause in-hospital mortality was used as the primary outcome of the study.

Patients eligible for CCP treatment had severe COVID-19 and a positive SARS-CoV-2 PCR test from the nasopharynx or by deep suction. The symptoms had to be present not more than 10 days and PCR diagnosis not more than 8 days. The definition of severe COVID-19 was: room air O_2_ saturation <94% and lung infiltrates involving >50% of the lung fields on chest X-ray or computed tomography. The study (Protocol ASF-0117-20) was approved by the hospital institutional review board (IRB). Informed consent was obtained from all patients. Patients under age of 18 years and those with active bacterial infection requiring antibiotic treatment were excluded.

Magen David Adom National Blood Services in Israel collected plasma by plasmapheresis from recovered COVID-19 patients with high titer anti-SARS-CoV-2 IgG antibodies. Donors aged between 18 and 65 years tested negative for SARS-CoV-2 at the time of the procedure and provided written informed consent. If donors were found eligible according to standard blood donor criteria, they were recruited in a plasmapheresis CCP collection program.

Two 200ml units of CCP were administered within 24 hours of each other to COVID-19 patients with severe COVID-19 pneumonia.

### Donors’ testing

CCP donations were tested for anti-N by CMIA, performed on the Architect i2000 SR (Abbott, Green Oaks, IL) [14]. Result of sample/cut-off (S/CO) ≥1.4 was defined as positive, high titer S/CO≥4 [15]. Performance of this test was evaluated and a specificity of more than 99% was observed [16].

Of the two CCP units administered, unit one had an median antibody concentration of S/CO 5.92 and unit two S/CO 3.36. The median Ab level of S/CO was 4.61, in accordance with the FDA decision [17].

### Data collection

Age, sex, origin (Jewish or Moslem), living at home or in an institution, smoking status, comorbidities, laboratory tests, chest X-ray, treatments and survival status at discharge, were recorded. The comorbidities included in the study were hypertension, diabetes mellitus, chronic ischemic heart disease, atrial fibrillation, chronic renal failure, chronic dialysis, kidney transplant, acute renal failure and dialysis, chronic obstructive lung disease, solid tumors, hemato-oncologic diseases, cerebrovascular diseases, neurologic disease, hypothyroidism and cirrhosis. Basic metabolic index, Morse fall risk, Norton bedsore risk and Mews scores were obtained. Patients’ ABO and RhD blood groups, complete blood count, neutrophil to lymphocyte ratio (NLR), urea, creatinine level, estimated GFR, albumin, bilirubin, alkaline phosphatase, alanine aminotransferase, aspartate aminotransferase, lactic dehydrogenase, C-reactive protein (CRP), ferritin, D-dimer and SO_2_ saturation in room air were extracted. All data was collected from the patients’ electronic health records (EHR). Patients with mechanical ventilation were censored on admission and at treatment. Additional treatments were administered including: Dexamethasone 6mg (IV or PO) for 10 days to 98 patients, Remdesivir 400mg IV on day 1 and 100mg on days 2–5 to 81 patients, Tociluzumab 400mg IV for 2 days to 61 patients and Lopinavir-Ritonavir 200mg PO twice daily to 2 patients.

### Statistical methods

Categorical variables were summarized as frequency and percentage. Continuous variables were evaluated to meet the normal distribution using histogram and Kolmogorov Smirnov test. Normally distributed continuous variables were reported as mean and standard deviation, while other variables were reported as median and interquartile range. Categorical variables were compared between those who died during hospitalization and those who survived using chi-squared test or Fisher’s exact test. Continuous variables were compared using independent sample t-test or Mann-Whitney test. Variables that were significantly associated with in-hospital mortality in the univariate analysis were included in the multivariable analysis. Multivariable logistic regression using backward methods was applied to identify independent predicators for in-hospital mortality. Wald test with a P-value of >0.1 was used as criteria for variable removal. All statistical tests were two sided and P<0.05 was considered statistically significant. SPSS software was used for all statistical analyses (IBM SPSS statistics for windows, version 25, Armonk, NY, USA 2017).

## Results

One hundred and twelve patients with severe COVID-19 received CCP. Median time from admission to hospital until plasma transfusion was 1 day (IQR 1–3.8). Median age was 67 years (IQR 55–74) and 66 (58.9%) were males. The patients’ characteristics, morbidities, laboratory results and univariate analysis of them are presented in Table 1.

**Table 1:** Patients receiving convalescent Covid plasma.

In-hospital mortality				
	Total	Alive	Dead	p
Population, n (%)	112	92	20	
Male, n (%)	66 (58.9)	52 (56.5)	14 (70)	0.267
Age, median (IQR)	67.5 (55.3–74)	64.5 (53–72)	73.5 (69.3–78.6)	<0.001
Jewish, n (%)	89 (79.5)	71 (77.2)	18 (90)	0.24
Moslem, n (%)	23 (20.5)	21 (22.8)	2 (10)	
Live at home, n (%)	106 (94.6)	87 (94.6)	19 (95)	>0.999
Smoker, n (%)	11 (9.8)	9 (9.8)	2 (10)	>0.999
Basic metabolic index, median (IQR)	30 (27–33)	30 (27–34)	30 (25–32)	0.49
Morse fall risk, median (IQR)	25 (14–40)	20 (15–38)	40 (24–54)	0.003
Norton bedsore risk, median (IQR)	22 (20–23)	22 (20–23)	18 (22–23)	0.08
MEWS score, median (IQR)	6 (5–7)	5 (6–7)	7 (6–8)	0.13
Bilateral pneumonia on X-Ray on admission, n (%)	92 (82.1)	76 (82.6)	16 (80)	0.745
Ventilation, n (%)	18 (16.1)	5 (5.4)	13 (65)	<0.001
Blood Group (ABO), n (%)				0.287
O	27 (24.1)	25 (27.2)	2 (10)	
A	52 (46.4)	42 (45.7)	10 (50)	
AB	10 (8.9)	7 (7.6)	3 (15)	
B	23 (20.5)	18 (19.6)	5 (25)	
Blood Group (RhD), n (%)				>0.999
RhD positive	104 (92.9)	85 (92.4)	19 (95)	
RhD negative	8 (7.1)	7 (7.6)	1 (5)	
Comorbidities, n (%)				
Hypertension	65 (58)	46 (50)	19 (95)	<0.001
Diabetes Mellitus	52 (46.4)	34 (37)	18 (90)	<0.001
Chronic ischemic heart disease	31 (27.7)	21 (22.8)	10 (50)	0.02
Congestive heart failure	7 (6.3)	5 (5.4)	2 (10)	0.613
Atrial fibrillation	6 (5.4)	5 (5.4)	1 (5)	>0.999
Chronic renal failure	14 (12.5)	6 (6.5)	8 (40)	0.001
Dialysis	1 (0.9)	0	1 (5)	0.179
Kidney transplant	2 (1.8)	1 (1.1)	1 (5)	0.327
Acute renal failure dialysis	1 (0.9)	0	1 (5)	0.179
Chronic obstructive lung disease/asthma	11 (9.8)	9 (9.8)	2 (10)	>0.999
Solid tumor	10 (8.9)	7 (7.6)	3 (15)	0.38
Hemato-oncology	4 (3.6)	2 (2.2)	2 (10)	0.146
Cerebro vascular disease	5 (4.5)	5 (5.4)	0	0.583
Neurologic disease	3 (2.7)	2 (2.2)	1 (5)	0.449
Hypothyroidism	2 (1.8)	2 (2.2)	0	>0.999
Cirrhosis	1 (0.9)	0	1 (5)	0.179
None	30 (26.8)	30 (32.6)	0	0.003
Time to plasma transfusion, days, median (IQR)	1 (1–3.8)	1 (1–3)	2 (1–4.8)	0.498
Treatment, n (%)				
Kaletra	2 (1.8)	2 (2.2)	0	>0.999
Tociluzumab	61 (54.5)	55 (59.8)	6 (30)	0.015
Remdesivir	81 (72.3)	66 (71.7)	15 (75)	0.878
Dexamethasone	98 (87.5)	79 (85.9)	19 (95)	0.458
Admission values^a^				
SO_2_%, median (IQR)	95 (92–97)	96 (94–97)	92 (90–95)	0.001
Anemia^b^, number (%)	34 (30.4)	22 (23.9)	12 (60)	0.001
Absolute white blood cell count 10^3^/μL, median (IQR)	5.8 (4.4–7.4)	5.8 (4.5–7.0)	5.6 (3.8–10.2)	0.696
Absolute neutrophil count 10^3^/μL, median (IQR)	4.05 (3–5.6)	4.1 (3.2–5.4)	4.1 (2.8–8.4)	0.643
Absolute lymphocyte count 10^3^/μL, median (IQR)	0.9 (0.7–1.2)	0.9 (0.7–1.2)	0.7 (0.6–1.2)	0.307
Platelets 10^3^/μL, median (IQR)	174 (133–224)	176 (143–224)	156 (116–227)	0.293
Urea, median (IQR)	33.9 (26–47)	32 (23.8–41.5)	61 (38.2–94.8)	0.001
Creatinine, median (IQR)	0.88 (0.72–1.2)	0.86 (0.69–1)	1.5 (0.82–1.86)	0.001
eGFR, median (IQR)	80 (54–100)	84.41 (63.58–102.84)	46.52 (33.1–81.92)	0.001
Bilirubin, median (IQR)	0.45 (0.31–0.58)	0.45 (0.31–0.63)	0.45 (0.23–0.5)	0.636
Albumin, median (IQR)	36 (34–40)	36 (34–40)	33 (32–35)	0.002
Alkaline phosphatase, median (IQR)	67 (48–72)	66 (49–74)	73 (48–94)	0.397
Alanine amino transferase, median (IQR)	26 (17–40)	27 (18–40)	21 (17–47)	0.443
Aspartate amino transferase, median (IQR)	38 (27–49)	38 (27–48)	39 (24–56)	0.865
Lactic dehydrogenase, median (IQR)	672 (530–844)	666 (530–834)	672 (515–988)	0.684
C-reactive protein, median (IQR)	80.03 (39.54–147.28)	73.95 (35.88–146.21)	92 (55.83–235.45)	0.11
Ferritin, median (IQR)	750 (250–1288)	723 (250–1194)	997 (250–1632)	0.397
D-Dimer, median (IQR)	711 (518–1080)	675 (489–944)	1128 (712–2331)	0.014
Treatment values^a^				
SO_2_%, median (IQR)	90 (88–92)	90 (88–92)	89 (86–92)	0.202
Anemia^b^, number (%)	46 (41.1)	34 (37)	12 (60)	0.058
Absolute white blood cell count 10^3^/μL, median (IQR)	5.8 (4.5–8.2)	5.8 (4.6–7.6)	7.6 (3.9–13.3)	0.002
Absolute neutrophil count 10^3^/μL, median (IQR)	4.2 (3–6.8)	4.2 (3.1–6.1)	6.8 (2.9–12.4)	0.15
Absolute lymphocyte count 10^3^/μL, median (IQR)	0.9 (0.6–1.2)	0.9 (0.7–1.2)	0.7 (0.4–1.1)	0.019
Platelets 10^3^/μL, median (IQR)	177 (147–233)	178 (150–233)	166 (119–210)	0.258
Urea, median (IQR)	35 (24.7–47.2)	32 (23–45)	58 (37–94)	0.05
Creatinine, median (IQR)	0.87 (0.66–1.19)	0.86 (0.66–1.05)	1.42 (0.75–2.3)	0.006
eGFR, median (IQR)	83 (50–103)	85.79 (65.59–105.74)	47.83 (27.18–93.25)	0.009
Bilirubin, median (IQR)	0.44 (0.28–0.61)	0.44 (0.28–0.61)	0.45 (0.34–0.62)	0.727
Albumin, median (IQR)	35 (32–37)	36 (33–39)	32 (30–35)	0.002
Alkaline phosphatase, median (IQR)	62 (47–82)	61 (47–74)	65 (46–98)	0.375
Alanine amino transferase, median (IQR)	27 (17–43)	27 (17–40)	27 (17–49)	0.842
Aspartate amino transferase, median (IQR)	39 (27–51)	37 (26–51)	43 (33–50)	0.133
Lactic dehydrogenase, median (IQR)	707 (541–896)	700 (538–873)	806 (545–1017)	0.246
C-reactive protein, median (IQR)	119.55 (68.97–186.33)	110.9 (60.12–178.77)	151.54 (73.22–282.53)	0.07
Ferritin, median (IQR)	759 (327–1415)	751 (325–1358)	997 (334–2572)	0.261
D-Dimer, median (IQR)	742 (521–1204)	681 (481–1095)	1214 (720–2206)	0.008

^a^S1 Table. Normal laboratory values.

^b^Anemia = Hb<13.5g/dL males, Hb<12g/dL females.

Twenty (17.9%) of the 112 patients died. Fifty-two (46.4%) of the 112 patients in the study had diabetes, 34 (65.4%) of the diabetic patients survived and 18 (34.6%) of them died from COVID-19.

On univariate analysis the following parameters were statistically significantly associated with increased in-hospital mortality: diabetes mellitus, O_2_ saturation in room air on admission, mechanical ventilation, lower albumin levels, older age, higher Morse Fall Scale, decreased estimated GFR and presence of hypertension. Multivariable analysis was performed on these 8 variables, of them diabetes mellitus (p = 0.004, OR 91.54, 95% CI 4.1–2023.9), mechanical ventilation (p = 0.001, OR 59.07, 95% CI 5.5–629) and lower albumin levels at treatment (p = 0.027, OR 0.74, 95% CI 0.56–0.97) had a statistically significant association with increased in-hospital mortality.

The association between titer doses of both units administered (unit 1, unit 2), the mean titer of the two doses administered, time to treatment and in-hospital mortality, ventilation and ventilation or mortality was studied (Table 2). No significant differences were observed in any of the parameters.

**Table 2:** Antibody levels, days to treatment and in-hospital mortality, ventilation.

**antibody levels, S/C median (IQR)**	**cohort**		
**unit 1**	5.92 (4.59–7.18)		
**unit 2**	3.36 (2.38–5.11)		
**median**	4.61 (3.65–6.09)		
**days to treatment** ^**a**^	1 (1–3.75)		
	**in-hospital mortality**	**alive**	**p**
**unit 1**	5.92 (4.39–7.11)	5.91 (4.61–7.2)	0.981
**unit 2**	3.04 (1.98–5.2)	3.49 (2.39–5.05)	0.574
**median**	4.67 (3.29–6.09)	4.53 (3.65–6.08)	0.839
**days to treatment** ^**a**^	2 (1–5.5)	1 (1–3)	0.259
	**ventilation**	**no ventilation**	**p**
**unit 1**	5.78 (4.41–7.08)	5.99 (4.61–7.18)	0.802
**unit 2**	3.39 (2.09–4.87)	3.32 (2.38–5.13)	0.980
**median**	4.41 (3.48–6.1)	4.65 (3.64–6.08)	0.835
**days to treatment** ^**a**^	2 (0.75–6)	1 (1–3)	0.371
	**ventilation or mortality**	**no ventilation or mortality**	**p**
**unit 1**	5.87 (4.4–7.08)	5.96 (4.61–7.2)	0.852
**unit 2**	3.38 (2.03–5.14)	3.34 (2.38–5.11)	0.771
**median**	4.57 (3.38–5.95)	4.61 (3.65–6.09)	0.840
**days to treatment** ^**a**^	2 (1–5.25)	1 (1–3)	0.339

^a^median (IQR).

## Discussion

The COVID-19 pandemic is still present worldwide. New waves of the disease are occurring, even in countries where the population has been vaccinated, due to the emergence of new variants and the decreasing efficacy of existing vaccines. Therefore, patients with a high risk of morbidity and mortality should be identified early on in order to administer the best combination of treatments available and being approved for this severe disease. This study analyzes outcome in 112 severe COVID-19 patients who received two units of CCP, at least one of them high titer (Table 2) in the early stages of their disease during the first COVID-19 wave in Israel. Three parameters were associated with in-hospital mortality on multivariable analysis, diabetes (p = 0.004, OR 91.54), mechanical ventilation (p = 0.001, OR 59.07) and lower albumin levels on admission (p = 0.027, OR 0.74).

CCP therapy has been used in the treatment of infectious diseases including SARS-CoV-1, CoV-MERS and H1N1 (2009) [18, 19]. The use of passive antibody transfer was considered as possible treatment for COVID-19 patients and early on in the pandemic an emergency use authorization for CCP was issued by the FDA [20]. Different doses of CCP have been administered in various protocols [8, 18, 21, 22].

Korley et al. did not demonstrate a reduction of mortality in COVID-19 patients treated with CCP [23]. The Cochrane living systemic review for CCP or hyperimmune immunoglobulin treatment for people with COVID-19 was very uncertain about the effect of convalescent plasma on all‐cause mortality (RR 0.50, 95% CI 0.09 to 2.65; very low‐certainty evidence), and inconclusive about the effect of convalescent plasma on developing severe clinical COVID‐19 symptoms (RR not estimable; low‐certainty evidence) [24]. The REMAP-CAP trial primary analysis included 1075 critically ill COVID-19 patients who received CCP, 11 patients who received delayed CCP and 904 patients who did not receive CCP. There was no statistically significant difference in in-hospital mortality between the groups [25]. In the RECOVERY trial, 11,558 hospitalized patients were randomized either to receive CCP (5,795) or the standard care (5,763), the conclusion was that high-dose CCP did not improve survival. However, they stated with caution that the possibility of small improvements in the probability of successful discharge from hospital by day 28 or of progressing to invasive mechanical ventilation or death in seronegative patients who received convalescent plasma could not be excluded [26]. However, other studies have demonstrated reduced mortality in in-hospital patients treated with CCP. The ConPlas-19 study in Spain included 350 patients and showed a significant benefit in preventing progression to noninvasive ventilation or high-flow oxygen, invasive mechanical ventilation or ECMO, or death at 28 days [27]. O’Donnell et al. conducted a double blind, randomized control trial among critical and severe COVID-19 hospitalized patients in New-York and in Brazil. The patients were randomized 2:1 to receive either CCP or normal control plasma. Twenty-eight-day mortality was significantly lower in participants randomized to convalescent plasma versus control plasma (19/150 [12.6%] versus 18/73 [24.6%], OR 0.44, 95% CI 0.22–0.91, *P* = 0.034) [28]. In the CAPSID study, 105 hospitalized patients in Germany were randomized to receive standard treatment and CCP or standard treatment alone. Primary endpoints, including survival and no longer fulfilling the criteria for severe COVID-19 were not improved, however there was a significant benefit in a subgroup of patients who received a larger amount of neutralizing antibodies [29]. In order to reduce the risk of progression of disease resulting in hospitalization, Sullivan et al. examined the administration of CCP to outpatients, and observed that within 9 days after the onset of symptoms, there was a reduced risk of disease progression leading to hospitalization, most of the patients were unvaccinated [30]. Furthermore, evidence points to a possible role for this treatment in immunocompromised patients, a subgroup at risk for severe and prolonged COVID-19 [11–13]. Kenig et al. reported 8 patients presenting with prolonged disease course and delayed viral clearance. They received CCP in addition to standard medical treatment, all patients showed remarkable clinical and laboratory improvement with rapid conversion of polymerase chain reaction from nasopharyngeal swabs to negative post CCP treatment [31]. In a single center series hematological immunocompromised patients received CCP for persistent COVID-19 symptoms, it contributed to clinical and radiological improvement and recovery, with viral clearance in a subset of patients [32]. Fourteen patients with acquired immunodeficiency active COVID-19, with negative serology to SARS-CoV-2 received CCP. Thirteen developed anti-SARS-CoV-2 within 24–48 hours, 8 showed clinical improvement on day 5 and 12 were discharged from the hospital [33].

In a summary of 278 cases from 3 studies reviewing mortality from COVID-19 in Wuhan, China between December 2019 and March 2020 the clinical characteristics of the non-survivors included adult males, age older than 50 years and comorbidities included diabetes [34]. Since the onset of the epidemic diabetes has been described as a predictor of mortality. In a case series from Wuhan, China which examined 168 COVID-19 patients who died, diabetes was the second most common comorbidity observed in 42 patients (25%), the most common being hypertension in 84 patients (50%) and the least common ischemic heart disease in 31 patients (18.5%) [35]. In a study that analyzed mortality causes among 44,672 cases of COVID-19, there were 1,073 mortalities (2.3%) and among the diabetics there was a high case fatality rate (7.3%) [36]. A meta-analysis by Varikasuvu et al. demonstrated that COVID-19 patients with diabetes have a significantly higher risk of disease severity (OR = 2.20, 95% CI = 1.69–2.86, Z = 5.82, p < 0.00001) and associated mortality outcomes (OR = 2.52, 95% CI = 1.93–3.30, Z = 6.79, p = < 0.00001) [37]. In our study, there were 52 patients with COVID-19 and diabetes, 18 of them (16% of the cohort, 90% of deaths) died, the difference was statistically significant on univariate (p<0.001) and multivariable (p = 0.004) analysis. There were 60 patients without diabetes, 58 (96.7%) of them survived and 2 (3.3%) died, the difference between mortality in the cohort with diabetes, compared to that without was statistically significant (p<0.001).

Angiotensin converting enzyme 2 (ACE2), which is the functional host receptor for SARS-CoV-2 [38], may be partially responsible for the severity of the disease in diabetics. The increase of ACE2 in pneumocytes [39] can be associated with changes in severity of COVID-19 in diabetic patients compared to non-diabetics, resulting in higher mortality in diabetic subjects [40]. The higher ACE2 expression in the lungs could enable SARS-CoV-2 entry and replication in diabetics, is possibly related to severity of COVID-19, helping to explain worse outcomes in this group of patients [41]. ACE2 expression is crucial to activate anti-inflammatory and anti-fibrotic processes in the lung and to maintain the vascular integrity of lung blood vessels [42]. Another route of infection is via transmembrane protease serine 2 (TMPRSS2) driven cleavage of SARS-CoV-2 escorted through ACE2 [43]. Thus, ACE2 overexpression in diabetic patients may possibly provide an insight into the statistically significant increased mortality in diabetics in general and in our cohort of COVID-19 patients specifically despite treatment with CCP early on in their disease. On the other hand, lower levels of ACE-2 may influence severity of COVID-19, possibly via the renin-angiotensin-aldosterone system [44].

As we stated above, apart from diabetes, mechanical ventilation could also serve as predictor of mortality. In our study 18 patients required mechanical ventilation, of these 13 (died (13/20), 65% of all deaths), this finding was statistically significant on multivariable analysis. Globally, the case fatality rate for severe COVID-19 patients admitted to the intensive care units and receiving invasive mechanical ventilation is high as shown in a systemic review and meta-analysis of 69 studies with 45% (95% CI 39–52%) mortality across all studies [45]. A study from Northern Virginia, USA of 1,023 COVID-19 patients admitted to hospital, included 164 (16%) patients that required invasive mechanical ventilation, of them 70 (42.7%) died [46]. We assume that the patients who required mechanical ventilation in our study were admitted with severe COVID-19 in the first place and belong a priory to a group with a worse prognosis. In order to see if antibody levels and time to treatment had an effect on in-hospital mortality in ventilated or non-ventilated patients, we analyzed the following variables according to antibody level and time to treatment: ventilation, in-hospital mortality and ventilation or in-hospital mortality. No statistical significance was observed (Table 2).

In accordance with previous studies, we acknowledge the risk factor low albumin level to be associated with increased mortality. Tanboga et al. developed and validated prediction models to identify in-hospital deaths in COVID-19 patients using predictors measured on admission in all hospitalized patients [47]. Lower albumin levels were found on multivariable analysis to be one of the strongest predictors of 30-day mortality [OR 0.34 (95% CI 0.26–0.45)]. Kashefizadeh et al. described 53 COVID-19 patients admitted to hospital between March and April 2020, in their study lower levels of albumin were associated with mortality p = 0.025 (OR 0.036 95% CI 0.002–0.655) [48]. Lower serum albumin concentrations were also found to be significantly associated with disease severity and adverse outcomes in COVID-19 patients p<0.001 [SMD -0.99 (95% CI -1.11 to -0.88)] by Paliogiannis et al., suggesting that assessment of serum albumin concentrations might assist with early risk stratification and selection of appropriate care pathways in severe COVID-19 patients with a poor outcome [49]. In a meta-analysis of 7 studies, albumin levels were lower on admission in non-survivors compared to survivors (−3.7 g/L, 95% CI, −5.3 to −2.1; P<0.00001) [50]. A study analyzing the relationship between albumin and survival in 319 COVID-19 patients found that this parameter was independently associated with mortality p<0.001 [HR 0.38, (95% CI 0.23–0.63)], and concluded that albumin may help in the early identification of patients at higher mortality risk in this disease [51]. In our study, as lower albumin levels on admission were also significantly associated with mortality and in accordance with these reports, we assume that it may be considered a marker useful for early assessment of severe disease, possibly explaining the worse prognosis in our cohort.

The association of autoantibodies and severe COVID-19 has been studied and they have been suggested as prognostic serological markers. Pascolini et al. studied 33 consecutive patients with COVID-19, 31 of them with interstitial pneumonia [52]. Fifteen of 33 patients (45%) had at least one autoantibody and some had multiple antibodies including ANA, anticardiolipin, antibeta2glycoprotein 1. Six of the fifteen patients (40%) died of complications of COVID-19. They suggested that patients with severe COVID-19 had immune dysregulation, possibly associated with a poor prognosis. Muratori et al. had a similar observation [53].

The study had several limitations. The data described is a single center experience. However, the medical center is an 891 beds acute care hospital serving a large urban, rural and multiethnic population of more than 1.5 million people. The data was collected during the first 5 months of the COVID-19 pandemic in Israel before the introduction of vaccinations. However, the study’s results may be relevant in populations with low vaccination percentages as the study population was unvaccinated. During the study period all severe COVID-19 patients received CCP, therefore a cohort of patients who didn’t receive this treatment was not available to make an outcome comparison. We analyzed the data according to antibody titer in each of the two units of CCO administered, the mean titer of the two units, time to treatment and the effect of these variables on in-hospital mortality, ventilation and mortality or ventilation and found no statistical difference. We cannot comment on the prognostic significance of autoantibodies, as we didn’t search for them in our cohort.

## Conclusions

Diabetes, mechanical ventilation and lower albumin levels on admission were statistically significant predictors of in-hospital mortality in the cohort of patients who received CCP. This has been described in the general COVID-19 population, including patients who did not receive CCP. As evidence points toward a benefit from CCP treatment in immunocompromised patients, we believe the above risk factors can further define COVID-19 patients at increased risk for mortality, enabling the selection of candidates for early treatment, in an outpatient setting if possible.

## Supporting information

S1 Table Normal laboratory values. (DOCX)

## References

[1] Parums D V. Editorial: Current Status of Oral Antiviral Drug Treatments for SARS-CoV-2 Infection in Non-Hospitalized Patients. Vol. 28, Medical science monitor: international medical journal of experimental and clinical research. 2022. p. e935952. doi: 10.12659/MSM.935952 34972812 PMC8729033

[2] Stinebaugh BJ, Schloeder FX, Johnson KM, Mackenzie RB, Entwisle G, De Alba E. Bolivian hemorrhagic fever: A report of four cases. Am J Med [Internet]. 1966 Feb 1;40(2):217–30. Available from: doi: 10.1016/0002-9343(66)90103-3 4159195

[3] Mupapa K, Massamba M, Kibadi K, Kuvula K, Bwaka A, Kipasa M, et al. Treatment of Ebola hemorrhagic fever with blood transfusions from convalescent patients. International Scientific and Technical Committee. J Infect Dis. 1999 Feb;179 Suppl:S18–23. doi: 10.1086/514298 9988160

[4] Mair-Jenkins J, Saavedra-Campos M, Baillie JK, Cleary P, Khaw F-M, Lim WS, et al. The effectiveness of convalescent plasma and hyperimmune immunoglobulin for the treatment of severe acute respiratory infections of viral etiology: a systematic review and exploratory meta-analysis. J Infect Dis. 2015 Jan;211(1):80–90. doi: 10.1093/infdis/jiu396 25030060 PMC4264590

[5] Cheng Y, Wong R, Soo YOY, Wong WS, Lee CK, Ng MHL, et al. Use of convalescent plasma therapy in SARS patients in Hong Kong. Eur J Clin Microbiol Infect Dis Off Publ Eur Soc Clin Microbiol. 2005 Jan;24(1):44–6. doi: 10.1007/s10096-004-1271-9 15616839 PMC7088355

[6] Focosi D, Franchini M, Pirofski L-A, Burnouf T, Fairweather D, Joyner MJ, et al. COVID-19 Convalescent Plasma Is More than Neutralizing Antibodies: A Narrative Review of Potential Beneficial and Detrimental Co-Factors. Viruses. 2021 Aug;13(8). doi: 10.3390/v13081594 34452459 PMC8402718

[7] Rajendran K, Krishnasamy N, Rangarajan J, Rathinam J, Natarajan M, Ramachandran A. Convalescent plasma transfusion for the treatment of COVID-19: Systematic review. J Med Virol. 2020 Sep;92(9):1475–83. doi: 10.1002/jmv.25961 32356910 PMC7267113

[8] Li L, Zhang W, Hu Y, Tong X, Zheng S, Yang J, et al. Effect of Convalescent Plasma Therapy on Time to Clinical Improvement in Patients With Severe and Life-threatening COVID-19: A Randomized Clinical Trial. JAMA. 2020 Aug;324(5):460–70. doi: 10.1001/jama.2020.10044 32492084 PMC7270883

[9] Joyner MJ, Wright RS, Fairweather D, Senefeld JW, Bruno KA, Klassen SA, et al. Early safety indicators of COVID-19 convalescent plasma in 5000 patients. J Clin Invest. 2020 Sep;130(9):4791–7. doi: 10.1172/JCI140200 32525844 PMC7456238

[10] Joyner MJ, Bruno KA, Klassen SA, Kunze KL, Johnson PW, Lesser ER, et al. Safety Update: COVID-19 Convalescent Plasma in 20,000 Hospitalized Patients. Mayo Clin Proc. 2020 Sep;95(9):1888–97. doi: 10.1016/j.mayocp.2020.06.028 32861333 PMC7368917

[11] Thompson MA, Henderson JP, Shah PK, Rubinstein SM, Joyner MJ, Choueiri TK, et al. Association of Convalescent Plasma Therapy With Survival in Patients With Hematologic Cancers and COVID-19. JAMA Oncol. 2021 Jun;7(8):1167–75. doi: 10.1001/jamaoncol.2021.1799 34137799 PMC8377563

[12] Betrains A, Godinas L, Woei-A-Jin FJSH, Rosseels W, Van Herck Y, Lorent N, et al. Convalescent plasma treatment of persistent severe acute respiratory syndrome coronavirus-2 (SARS-CoV-2) infection in patients with lymphoma with impaired humoral immunity and lack of neutralising antibodies. Vol. 192, British journal of haematology. England; 2021. p. 1100–5. doi: 10.1111/bjh.17266 33314018

[13] Fung M, Nambiar A, Pandey S, Aldrich JM, Teraoka J, Freise C, et al. Treatment of immunocompromised COVID-19 patients with convalescent plasma. Transpl Infect Dis. 2021 Apr;23(2):e13477. doi: 10.1111/tid.13477 32989856 PMC7537112

[14] Manalac J, Yee J, Calayag K, Nguyen L, Patel PM, Zhou D, et al. Evaluation of Abbott anti-SARS-CoV-2 CMIA IgG and Euroimmun ELISA IgG/IgA assays in a clinical lab. Clin Chim Acta. 2020 Nov;510:687–90. doi: 10.1016/j.cca.2020.09.002 32910980 PMC7476889

[15] Meschi S, Colavita F, Bordi L, Matusali G, Lapa D, Amendola A, et al. Performance evaluation of Abbott ARCHITECT SARS-CoV-2 IgG immunoassay in comparison with indirect immunofluorescence and virus microneutralization test. J Clin Virol Off Publ Pan Am Soc Clin Virol. 2020 Aug;129:104539. doi: 10.1016/j.jcv.2020.104539 32679298 PMC7336910

[16] Theel ES, Harring J, Hilgart H, Granger D. Performance Characteristics of Four High-Throughput Immunoassays for Detection of IgG Antibodies against SARS-CoV-2. J Clin Microbiol. 2020 Jul;58(8). doi: 10.1128/JCM.01243-20 32513859 PMC7383546

[17] FDA In Brief: FDA Updates Emergency Use Authorization for COVID-19 Convalescent Plasma to Reflect New Data. Available from: fda: revision of the Emergency Use Authorization (EUA) for COVID-19 convalescent plasma. https://www.fda.gov/news-events/fda-brief/fda-brief-fda-updates-emergency-use-authorization-covid-19-convalescent-plasma-reflect-new-data. Accessed 4 February 2021.

[18] Duan K, Liu B, Li C, Zhang H, Yu T, Qu J, et al. Effectiveness of convalescent plasma therapy in severe COVID-19 patients. Proc Natl Acad Sci U S A. 2020 Apr;117(17):9490–6. doi: 10.1073/pnas.2004168117 32253318 PMC7196837

[19] Soo YOY, Cheng Y, Wong R, Hui DS, Lee CK, Tsang KKS, et al. Retrospective comparison of convalescent plasma with continuing high-dose methylprednisolone treatment in SARS patients. Clin Microbiol Infect Off Publ Eur Soc Clin Microbiol Infect Dis. 2004 Jul;10(7):676–8. doi: 10.1111/j.1469-0691.2004.00956.x 15214887 PMC7129386

[20] FDA: Recommendations for Investigational COVID-19 Convalescent Plasma. Available from: https://www.fda.gov/vaccines-blood-biologics/investigational-new-drug-applications-inds-cber-regulated-products/recommendations-investigational-covid-19-convalescent-plasma. Accessed 13 July, 2021.

[21] Shen C, Wang Z, Zhao F, Yang Y, Li J, Yuan J, et al. Treatment of 5 Critically Ill Patients With COVID-19 With Convalescent Plasma. JAMA. 2020 Mar;323(16):1582–9. doi: 10.1001/jama.2020.4783 32219428 PMC7101507

[22] Libster R, Pérez Marc G, Wappner D, Coviello S, Bianchi A, Braem V, et al. Early High-Titer Plasma Therapy to Prevent Severe Covid-19 in Older Adults. N Engl J Med [Internet]. 2021 Jan 6;384(7):610–8. Available from: doi: 10.1056/NEJMoa2033700 33406353 PMC7793608

[23] Korley FK, Durkalski-Mauldin V, Yeatts SD, Schulman K, Davenport RD, Dumont LJ, et al. Early Convalescent Plasma for High-Risk Outpatients with Covid-19. N Engl J Med. 2021 Aug; doi: 10.1056/NEJMoa2103784 34407339 PMC8385553

[24] Piechotta V, Iannizzi C, Chai KL, Valk SJ, Kimber C, Dorando E, et al. Convalescent plasma or hyperimmune immunoglobulin for people with COVID-19: a living systematic review. Cochrane Database Syst Rev [Internet]. 2021 May 20 [cited 2021 Sep 21];2021(5). Available from: doi: 10.1002/14651858.CD013600.pub4 34013969 PMC8135693

[25] Estcourt LJ, Turgeon AF, McQuilten ZK, McVerry BJ, Al-Beidh F, Annane D, et al. Effect of Convalescent Plasma on Organ Support-Free Days in Critically Ill Patients With COVID-19: A Randomized Clinical Trial. JAMA. 2021 Nov;326(17):1690–702. doi: 10.1001/jama.2021.18178 34606578 PMC8491132

[26] Convalescent plasma in patients admitted to hospital with COVID-19 (RECOVERY): a randomised controlled, open-label, platform trial. Lancet (London, England). 2021 May;397(10289):2049–59. doi: 10.1016/S0140-6736(21)00897-7 34000257 PMC8121538

[27] Avendaño-Solá C, Ramos-Martínez A, Muñez-Rubio E, Ruiz-Antorán B, Malo de Molina R, Torres F, et al. A multicenter randomized open-label clinical trial for convalescent plasma in patients hospitalized with COVID-19 pneumonia. J Clin Invest. 2021 Oct;131(20). doi: 10.1172/JCI152740 34473652 PMC8516461

[28] O’Donnell MR, Grinsztejn B, Cummings MJ, Justman JE, Lamb MR, Eckhardt CM, et al. A randomized double-blind controlled trial of convalescent plasma in adults with severe COVID-19. J Clin Invest. 2021 Jul;131(13). doi: 10.1172/JCI150646 33974559 PMC8245169

[29] Körper S, Weiss M, Zickler D, Wiesmann T, Zacharowski K, Corman VM, et al. Results of the CAPSID randomized trial for high-dose convalescent plasma in patients with severe COVID-19. J Clin Invest. 2021 Oct;131(20). doi: 10.1172/JCI152264 34464358 PMC8516466

[30] Sullivan DJ, Gebo KA, Shoham S, Bloch EM, Lau B, Shenoy AG, et al. Early Outpatient Treatment for Covid-19 with Convalescent Plasma. N Engl J Med [Internet]. 2022 Mar 30;386(18):1700–11. Available from: doi: 10.1056/NEJMoa2119657 35353960 PMC9006786

[31] Kenig A, Ishay Y, Kharouf F, Rubin L. Treatment of B-cell depleted COVID-19 patients with convalescent plasma and plasma-based products. Clin Immunol. 2021 Jun;227:108723. doi: 10.1016/j.clim.2021.108723 33838340 PMC8024218

[32] Ferrari S, Caprioli C, Weber A, Rambaldi A, Lussana F. Convalescent hyperimmune plasma for chemo-immunotherapy induced immunodeficiency in COVID-19 patients with hematological malignancies. Leuk Lymphoma. 2021 Jun;62(6):1490–6. doi: 10.1080/10428194.2021.1872070 33461387 PMC7832449

[33] Rodionov RN, Biener A, Spieth P, Achleitner M, Hölig K, Aringer M, et al. Potential benefit of convalescent plasma transfusions in immunocompromised patients with COVID-19. The Lancet Microbe [Internet]. 2021;2(4):e138. Available from: https://www.sciencedirect.com/science/article/pii/S2666524721000306 doi: 10.1016/S2666-5247(21)00030-6 33817676 PMC8009633

[34] Elnour AA, Don J, Yousif I, Gnana K, Abdi S, Alhajri N, et al. The early mortality rate of people infected with coronavirus (COVID-2019) in Wuhan, China: Review of three retrospective studies. J Pharm Bioallied Sci. 2020;12(3):223–33. doi: 10.4103/jpbs.JPBS_282_20 33100781 PMC7574747

[35] Xie J, Tong Z, Guan X, Du B, Qiu H. Clinical Characteristics of Patients Who Died of Coronavirus Disease 2019 in China. JAMA Netw open. 2020 Apr;3(4):e205619. doi: 10.1001/jamanetworkopen.2020.5619 32275319 PMC7148440

[36] Wu Z, McGoogan JM. Characteristics of and Important Lessons From the Coronavirus Disease 2019 (COVID-19) Outbreak in China: Summary of a Report of 72 314 Cases From the Chinese Center for Disease Control and Prevention. JAMA. 2020 Apr;323(13):1239–42. doi: 10.1001/jama.2020.2648 32091533

[37] Varikasuvu SR, Dutt N, Thangappazham B, Varshney S. Diabetes and COVID-19: A pooled analysis related to disease severity and mortality. Prim Care Diabetes. 2021 Feb;15(1):24–7. doi: 10.1016/j.pcd.2020.08.015 32891525 PMC7456278

[38] Bourgonje AR, Abdulle AE, Timens W, Hillebrands J-L, Navis GJ, Gordijn SJ, et al. Angiotensin-converting enzyme 2 (ACE2), SARS-CoV-2 and the pathophysiology of coronavirus disease 2019 (COVID-19). J Pathol. 2020 Jul;251(3):228–48. doi: 10.1002/path.5471 32418199 PMC7276767

[39] Roca-Ho H, Riera M, Palau V, Pascual J, Soler MJ. Characterization of ACE and ACE2 Expression within Different Organs of the NOD Mouse. Int J Mol Sci. 2017 Mar;18(3). doi: 10.3390/ijms18030563 28273875 PMC5372579

[40] Jeong I-K, Yoon KH, Lee MK. Diabetes and COVID-19: Global and regional perspectives. Diabetes Res Clin Pract. 2020 Aug;166:108303. doi: 10.1016/j.diabres.2020.108303 32623038 PMC7332438

[41] Apicella M, Campopiano MC, Mantuano M, Mazoni L, Coppelli A, Del Prato S. COVID-19 in people with diabetes: understanding the reasons for worse outcomes. lancet Diabetes Endocrinol. 2020 Sep;8(9):782–92. doi: 10.1016/S2213-8587(20)30238-2 32687793 PMC7367664

[42] Dalan R, Bornstein SR, El-Armouche A, Rodionov RN, Markov A, Wielockx B, et al. The ACE-2 in COVID-19: Foe or Friend? Horm Metab Res = Horm und Stoffwechselforsch = Horm Metab. 2020 May;52(5):257–63. doi: 10.1055/a-1155-0501 32340044 PMC7339082

[43] Kumar P, Sah AK, Tripathi G, Kashyap A, Tripathi A, Rao R, et al. Role of ACE2 receptor and the landscape of treatment options from convalescent plasma therapy to the drug repurposing in COVID-19. Mol Cell Biochem. 2021 Feb;476(2):553–74. doi: 10.1007/s11010-020-03924-2 33029696 PMC7539757

[44] Goldin CJ, Vázquez R, Polack FP, Alvarez-Paggi D. Identifying pathophysiological bases of disease in COVID-19. Transl Med Commun [Internet]. 2020;5(1):15. Available from: doi: 10.1186/s41231-020-00067-w 32984543 PMC7506209

[45] Lim ZJ, Subramaniam A, Ponnapa Reddy M, Blecher G, Kadam U, Afroz A, et al. Case Fatality Rates for Patients with COVID-19 Requiring Invasive Mechanical Ventilation. A Meta-analysis. Am J Respir Crit Care Med. 2021 Jan;203(1):54–66. doi: 10.1164/rccm.202006-2405OC 33119402 PMC7781141

[46] King CS, Sahjwani D, Brown AW, Feroz S, Cameron P, Osborn E, et al. Outcomes of mechanically ventilated patients with COVID-19 associated respiratory failure. PLoS One. 2020;15(11):e0242651. doi: 10.1371/journal.pone.0242651 33227024 PMC7682899

[47] Tanboğa IH, Canpolat U, Çetin EHÖ, Kundi H, Celik O, Cağlayan M, et al. Development and Validation of Clinical Prediction Models to Estimate the Probability of death in Hospitalized Patients with COVID-19: Insights from a Nationwide Database. J Med Virol. 2021 Feb; doi: 10.1002/jmv.26844 33527474 PMC8014660

[48] Kashefizadeh A, Ohadi L, Golmohammadi M, Araghi F, Dadkhahfar S, Kiani A, et al. Clinical features and short-term outcomes of COVID-19 in Tehran, Iran: An analysis of mortality and hospital stay. Acta Biomed. 2020 Nov;91(4):e2020147. doi: 10.23750/abm.v91i4.10206 33525218 PMC7927529

[49] Paliogiannis P, Mangoni AA, Cangemi M, Fois AG, Carru C, Zinellu A. Serum albumin concentrations are associated with disease severity and outcomes in coronavirus 19 disease (COVID-19): a systematic review and meta-analysis. Clin Exp Med. 2021; 10.1007/s10238-021-00686-z PMC7842395 33511503

[50] Tian W, Jiang W, Yao J, Nicholson CJ, Li RH, Sigurslid HH, et al. Predictors of mortality in hospitalized COVID-19 patients: A systematic review and meta-analysis. J Med Virol. 2020 Oct;92(10):1875–83. doi: 10.1002/jmv.26050 32441789 PMC7280666

[51] Violi F, Cangemi R, Romiti GF, Ceccarelli G, Oliva A, Alessandri F, et al. Is Albumin Predictor of Mortality in COVID-19? Vol. 35, Antioxidants & redox signaling. United States; 2021. p. 139–42. doi: 10.1089/ars.2020.8142 32524832

[52] Pascolini S, Vannini A, Deleonardi G, Ciordinik M, Sensoli A, Carletti I, et al. COVID-19 and Immunological Dysregulation: Can Autoantibodies be Useful? Clin Transl Sci. 2021 Mar;14(2):502–8. doi: 10.1111/cts.12908 32989903 PMC7536986

[53] Muratori P, Lenzi M, Muratori L, Granito A. Antinuclear antibodies in COVID 19. Vol. 14, Clinical and translational science. 2021. p. 1627–8. doi: 10.1111/cts.13026 33932091 PMC8239781

