# Supplementary material for: Predictors of mortality in COVID-19 patients treated with convalescent plasma therapy
Source: PLoS One. 2022 Jul 19;17(7):e0271036. doi: 10.1371/journal.pone.0271036 (PMC9295964; doi:10.1371/journal.pone.0271036)
Supplement: S1 Table — (DOCX) [file pone.0271036.s001.docx]

S1 Table: Normal values of laboratory tests

| **Normal ranges** | | **Units** | **Variable** |
| --- | --- | --- | --- |
| **Female** | **Male or Male and Female** |  |  |
|  | 4-11 | 10^3^/μL | Absolute white blood cell count |
|  | 2-7.7 | 10^3^/μL | Absolute neutrophil count |
|  | 1-4 | 10^3^/μL | Absolute lymphocyte count |
|  | 14-450 | 10^3^/μL | Platelets |
|  | 20-45 | mg/dL | Urea |
| 0.5-0.9 | 0.7-1.2 | mg/dL | Creatinine |
|  | 0.2-1.2 | mg/dL | Bilirubin |
|  | 34-48 | g/L | Albumin |
|  | 39-117 | U/L | Alkaline phosphatase |
| 4-31 | 4-41 | U/L | Alanine amino transferase |
| 5-32 | 5-38 | U/L | Aspartate amino transferase |
|  | 240-480 | U/L | Lactic dehydrogenase |
|  | <0.3-5 | mg/dL | C-reactive protein |
| 13-150 | 30-400 | µg/mL | Ferritin |
|  | 50-500 | ng/mL FEU | D-Dimer |
